# Supplementary material for: The genome sequence of the Red-underwing Skipper, Spialia sertorius (Hoffmannsegg, 1804) (Lepidoptera: Hesperiidae)
Source: Wellcome Open Res. 2026 Aug 25;10:390. Originally published 2025 Jul 30. [Version 2] doi: 10.12688/wellcomeopenres.24648.2 (PMC12552837; doi:10.12688/wellcomeopenres.24648.2)
Supplement: Peer review history

# Reviewer report: Reviewer response for version 1

**DOI:** 10.21956/wellcomeopenres.27162.r135178

**Published:** 2025-10-24

**Authors:** Bin Zhang

**Affiliations:** 1 Qingdao Agricultural University, Qingdao, China

This data note presents a high-quality, chromosome-level genome assembly of *Spialia sertorius* (Red-underwing Skipper), a species of ecological and conservation interest in the family Hesperiidae. The assembly meets the Earth BioGenome Project (EBP) reference standards (6.C.Q40) and provides detailed sequencing, assembly, and curation workflows. By integrating PacBio HiFi long reads and Hi-C data, the authors successfully generated a haplotype-resolved assembly, with 99.92% of Haplotype 1 assigned to chromosomal pseudomolecules (including W/Z sex chromosomes). This work fills a critical gap in genomic resources for Hesperiidae and supports future research on butterfly phylogenomics, population genetics, and conservation. However, several minor issues related to sample representativeness, genomic detail, and functional context need to be addressed to maximize the manuscript’s utility.

 1. While the mitochondrial genome length (15.34 kb) is reported, key features are missing: (1) gene order (e.g., arrangement of protein-coding genes, rRNA, tRNA); (2) GC content of mitochondrial regions (e.g., control region vs. coding regions); (3) comparisons with mitochondrial genomes of congeneric (e.g., *S. orbifer*) or confamilial species. This limits its application in evolutionary studies (e.g., mitochondrial phylogenetics of Hesperiidae).

 2. The manuscript states the genome “will be annotated using available RNA-Seq data” but provides no preliminary results (e.g., number of predicted protein-coding genes, functional categories of BUSCO genes) or timelines for annotation release. Without even basic functional insights, it is impossible to link the assembly to *S. sertorius*’ unique characters (e.g., genes related to hostplant specialization on *Sanguisorba minor* or rapid flight behavior).

 3. While the authors reference *Thymelicus lineola* (GCA_963932265.1) for W/Z sex chromosome identification, no detailed comparison with *S. sertorius* is provided. Key evolutionary insights (e.g., synteny conservation, chromosome fusion/fission events) are not discussed, missed opportunities to contextualize the assembly within Lepidoptera genome evolution.

Are sufficient details of methods and materials provided to allow replication by others?

Yes

Is the rationale for creating the dataset(s) clearly described?

Yes

Are the datasets clearly presented in a useable and accessible format?

Yes

Are the protocols appropriate and is the work technically sound?

Yes

Reviewer Expertise:

Entomology and Bioinformatics

I confirm that I have read this submission and believe that I have an appropriate level of expertise to confirm that it is of an acceptable scientific standard.

# Reviewer report: Reviewer response for version 1

**DOI:** 10.21956/wellcomeopenres.27162.r135182

**Published:** 2025-10-24

**Authors:** Panagiotis Ioannidis

**Affiliations:** 1 Foundation for Research & Technology - Hellas, Crete, Greece

This manuscript describes the sequencing and assembly of the lepidopteran insect *Spialia sertorius*. All the methodology used for assembling is the standard one used by other Psyche/DToL genome projects and as such the resulting genome assembly is suitable for downstream analyses. This is demonstrated by the relatively low contig/scaffold to chromosome ratio (46/32=1.43), as well as the good Merqury QV and BUSCO scores.

 I should note here that the lack of a predicted gene set precludes the use of this assembly from a large number of researchers, because gene prediction is not trivial thing to do. Therefore, it would certainly be desirable to also have a predicted gene set (together with its BUSCO score!).

 I find it very interesting that chromosomes 27 and 28 have a considerably different GC% from the remaining chromosomes. Was this expected? Maybe the authors should briefly comment on it. And I think that such findings should be brought up in the text. I understand that this is a genome report, but in the same way that the authors (very appropriately) write details about the ecology and the life cycle of the insect, in that same sense they should also bring up "peculiar" findings from the analysis of their genome assembly. One sentence should suffice!

Are sufficient details of methods and materials provided to allow replication by others?

Yes

Is the rationale for creating the dataset(s) clearly described?

Yes

Are the datasets clearly presented in a useable and accessible format?

Yes

Are the protocols appropriate and is the work technically sound?

Yes

Reviewer Expertise:

insect genomics; bioinformatics

I confirm that I have read this submission and believe that I have an appropriate level of expertise to confirm that it is of an acceptable scientific standard.

# Reviewer report: Reviewer response for version 1

**DOI:** 10.21956/wellcomeopenres.27162.r128398

**Published:** 2025-08-29

**Authors:** Elena Pazhenkova

**Affiliations:** 1 University of Ljubljana, Ljubljana, Slovenia

The article presents a comprehensive genome assembly of the Red-underwing Skipper butterfly, *Spialia sertorius* (Lepidoptera, Hesperiidae). The authors have successfully constructed a high-quality assembly from a female specimen by utilizing Pacific Biosciences HiFi long-read sequencing and Hi-C data for scaffolding and phasing. The resulting assembly comprises two haplotypes, with total lengths of 364.57 megabases and 323.91 megabases. Notably, 99.92% of haplotype 1 is scaffolded into 32 chromosomal pseudomolecules, including both the W and Z sex chromosomes, while haplotype 2 was assembled to scaffold level. The mitochondrial genome, spanning 15.34 kilobases, has also been assembled.

 An estimated haploid genome size was 341.34 megabases, with a heterozygosity of 2.50% and a repeat content of 25.59%, providing a strong foundation for assembly expectations. High sequencing coverage for the nuclear genome, supplemented by extensive Hi-C data that facilitated chromosome-level assembly of haplotype, which was manually curated.  The chromosomal-level assembly represents a valuable genomic resource. It lays the groundwork for future research into the population genomics and evolutionary history of *S. sertorius*, especially within the context of its broad distribution and areas of sympatry with related species. The combination of extensive sequencing coverage, high contiguity, and careful curation makes this work a significant contribution to the field of butterfly genomics and biodiversity research.

Are sufficient details of methods and materials provided to allow replication by others?

Yes

Is the rationale for creating the dataset(s) clearly described?

Yes

Are the datasets clearly presented in a useable and accessible format?

Yes

Are the protocols appropriate and is the work technically sound?

Yes

Reviewer Expertise:

NA

I confirm that I have read this submission and believe that I have an appropriate level of expertise to confirm that it is of an acceptable scientific standard.
